# Multi-Scale Feature Fusion Convolutional Neural Network for Indoor Small Target Detection

**DOI:** 10.3389/fnbot.2022.881021

**Published:** 2022-05-19

**Authors:** Li Huang, Cheng Chen, Juntong Yun, Ying Sun, Jinrong Tian, Zhiqiang Hao, Hui Yu, Hongjie Ma

**Affiliations:** ^1^College of Computer Science and Technology, Wuhan University of Science and Technology, Wuhan, China; ^2^Hubei Province Key Laboratory of Intelligent Information Processing and Real-Time Industrial System, Wuhan University of Science and Technology, Wuhan, China; ^3^Key Laboratory of Metallurgical Equipment and Control Technology of Ministry of Education, Wuhan University of Science and Technology, Wuhan, China; ^4^Hubei Key Laboratory of Mechanical Transmission and Manufacturing Engineering, Wuhan University of Science and Technology, Wuhan, China; ^5^Precision Manufacturing Research Institute, Wuhan University of Science and Technology, Wuhan, China; ^6^School of Creative Technologies, University of Portsmouth, Portsmouth, United Kingdom; ^7^School of Energy and Electronic Engineering, University of Portsmouth, Portsmouth, United Kingdom

**Keywords:** indoor scene, small target detection, convolutional neural network, multi-scale feature fusion, SSD

## Abstract

The development of object detection technology makes it possible for robots to interact with people and the environment, but the changeable application scenarios make the detection accuracy of small and medium objects in the practical application of object detection technology low. In this paper, based on multi-scale feature fusion of indoor small target detection method, using the device to collect different indoor images with angle, light, and shade conditions, and use the image enhancement technology to set up and amplify a date set, with indoor scenarios and the SSD algorithm in target detection layer and its adjacent features fusion. The Faster R-CNN, YOLOv5, SSD, and SSD target detection models based on multi-scale feature fusion were trained on an indoor scene data set based on transfer learning. The experimental results show that multi-scale feature fusion can improve the detection accuracy of all kinds of objects, especially for objects with a relatively small scale. In addition, although the detection speed of the improved SSD algorithm decreases, it is faster than the Faster R-CNN, which better achieves the balance between target detection accuracy and speed.

## Introduction

With the development of the economy and technology, robots have become an integral piece of industrial equipment integrating machinery, control, and computer, and the level of their development has become another important standard to measure the scientific and technological level of countries (Jiang et al., 2019c, 2021b; Li et al., 2019a; Liu et al., 2021d). People's functional requirements for robots are not limited to one aspect or mechanization, such as programmatic operation, narrow human-machine interaction, etc., but instead robots are expected to realize intelligent operations according to the perception of the surrounding environment or the understanding of human voice and action instructions, that is, to realize intelligent interaction between machines and people and the environment (Huang et al., 2017; Li et al., 2019c, 2020; Portugal et al., 2019; Ma et al., 2020; Sun et al., 2020b, 2021). The emergence of computer vision makes it possible for robots to interact with the environment and people by understanding images like human beings (Jiang et al., 2019b; Chen et al., 2021a; Cheng et al., 2021). Target detection based on image recognition further determine the image of the object position, get the semantic information more comprehensively, and can define object category, location, the relationship between the various objects and images of the sensors and actuators, and scene semantic expression, to achieve an understanding of the scene (Brunetti et al., 2018; Tsai et al., 2018; Chen et al., 2022). This paper proposed an indoor small target detection method based on multi-scale feature fusion to improve the detection accuracy and detection speed of objects with different scales. In this paper, multi-scale features of SSD are studied, and multi-level features of SSD are not fully utilized, as SSD only uses a single-scale feature map to detect targets, which is not suitable for the actual multi-scale target application scenarios. The adjacent features of the fusion target detection layer are used to improve the network detection performance, and the multi-scale feature fusion structure is analyzed and designed. Finally, layer by layer features of SSD target detection layer are fused to obtain the optimal multi-scale feature fusion SSD target detection model.

The key contributions of this work are:

1. A literature survey about various existing target detection algorithm and an analysis of their advantages and disadvantages.
2. An indoor small target detection method based on multi-scale feature fusion is proposed, and the target detection layer and its adjacent feature layer are fused in the SSD algorithm.
3. Kinect is used to collect indoor color images under different angles, illumination, and occlusion, and image enhancement technology is used to amplify the data set to establish the data set under indoor scenes.
4. The performance of the proposed algorithm is analyzed and compared with other classical algorithms.

The rest of this paper is organized as follows: Section Related Work discusses related work, followed by target detection method based on multi-scale feature fusion in Section Multi-Scale Feature Fusion Convolutional Neural Network for Target Detection. Section Data Set Establishment and Experiment Based on Indoor Environment discusses the experiments and analyzes the results, and Section Conclusion concludes the paper with a summary and future research directions.

## Related Work

The task of target detection is to classify objects in an image and further determine their position in the image (Huang et al., 2019; Jiang et al., 2019a; Cheng et al., 2020; Liao et al., 2021; Hao et al., 2022). For the recognition task, the network needs to extract deeper semantic features, that is, the essence of the target features, so as to distinguish between the target objects and improve the accuracy of recognition. For positioning tasks, location information needs to be saved as much as possible to make the detection frame closer to the actual position of the target object in the image (Wang W. et al., 2017; Li et al., 2019b; Weng et al., 2021; Bai et al., 2022; Liu et al., 2022b; Tao et al., 2022b). The traditional target detection process proceeds as follows. Firstly, multiple image regions with possible target objects are selected by sliding windows of different sizes; then, feature extraction methods such as SIFT (Scale-invariant Feature Transform) (Raveendra and Vinothkanna, 2019) and HOG (Histogram of Oriented Gradient) (Zhou et al., 2018; Bilal and Hanif, 2019) transform the information contained in the region into feature vectors and then classify them. Support Vector Machine (SVM) (Seifi and Ghassemian, 2017; Xiang et al., 2017) classifier is commonly used. Viola and Jones (2004) discuss all possible positions of face features on the image through a sliding window and trained a detector that could detect faces of two people, completing real-time facial detection for the first time. However, the amount of calculation of the detector is too large and far exceeded the computing capacity at that time. DPM (Deformable Parts Model) is proposed; this model decomposes the target object into various parts for training, and merges the prediction results of all parts during prediction to complete the detection of the target object (Felzenszwalb et al., 2010). However, since the traditional target algorithm extracts the candidate region information and manually designs the features, the application range has great limitations (Lu et al., 2020). For example, the Haar feature is suitable for face detection, and the detector trained by this feature cannot detect other types of targets. In addition, the traditional target detection algorithm generates multiple candidate regions through traversal, which costs a lot of time. In addition, the traditional target detection algorithm classification training detector may produce the problem of feature vector “dimension disaster” (Zhao et al., 2019; Liao et al., 2020; Hao et al., 2021; Tao et al., 2022a; Zhang et al., 2022).

Hinton proposed deep learning to obtain the most representative features of images by learning network parameters. Ross et al. used Convolutional Neural Networks (CNN) to design the R-CNN object detection model, Selective Search (SS) is used to generate high-quality candidate regions on the image, AlexNet network is used to extract feature information, and SVM is used to obtain the target category and calibrate the detection box (Sharma and Thakur, 2017; Li et al., 2018). R-CNN uses depth for target detection for the first time, but the scaling of the candidate region has certain limitations on detection accuracy, and the training of this algorithm is also complicated. He et al. (2015) proposed SPP-NET, which can transform feature information of candidate regions of any size into feature vector of a fixed length. Felzenszwalb et al. (2010) uses ROI pooling (Region of Interest pooling) to fix the feature length of candidate areas and uses multi-task loss function for training. The algorithm of fast R-CNN greatly shortens the training and detection time of target detection algorithm. Faster R-CNN (Ren et al., 2015; Liu et al., 2021b) uses a network to generate candidate regions and shared weights, which enhances detection accuracy and speed. For the purpose of real-time detection, algorithms based on regression YOLO and SSD (Single Shot MultiBox Detector) have appeared successively (Li et al., 2017; Sun et al., 2020a; Liu et al., 2022c). The integrated convolutional neural network is used to complete target detection, thus improving the detection efficiency of the algorithm (Hu et al., 2019; Duan et al., 2021; Huang et al., 2021; Liu et al., 2021a). However, both SSD and YOLO only use the characteristic information of a single scale to predict, and the detection accuracy of multi-scale targets and small objects is low (Tian et al., 2020; Liu et al., 2021c, 2022a; Xiao et al., 2021). In order to improve the detection performance of small targets in various complex scenarios, researchers have carried out a series of studies, including feature fusion, context utilization, and adversarial learning. Xiang et al. (2017) proposed an inside-outside Network (ION) method. This method firstly cuts out candidate region features from different layers of the convolutional neural network, then normalizes feature regions of different scales by Region of Interest Pooling (RoI), and finally integrates these multi-scale features to improve regional feature expression ability. Multiple studies also attempt to integrate the context around the target into a deep neural network (Zeng et al., 2017). Furthermore, Wang et al. proposed an improved detection model based on Fast R-CNN for small target occlusion and deformation (Wang X. et al., 2017), which was trained from generated adversarial samples. In order to enhance the robustness to occlusion and deformation, a network which automatically generates occlusion and deformation features is introduced into the model (Yu et al., 2019, 2020; Zhao et al., 2021; Sun et al., 2022; Wu et al., 2022). The detection model can receive more adversarial samples through occlusion and deformation processing of regional features, so that the trained model has stronger capability.

But as a result of application scenarios and changes, such as light, the change of perspective will keep out problems such as objects have different scales will lead to target detection technology in the practical application under the effect not beautiful, intelligent service robot human-computer interaction exists degree is not high, difficult to meet the personalized requirements of users, problem, therefore, service-oriented robot application scenarios. How to improve the accuracy and real-time of object detection in complex environment is still challenging.

## Multi-Scale Feature Fusion Convolutional Neural Network for Target Detection

### SSD

**Figure 1:**
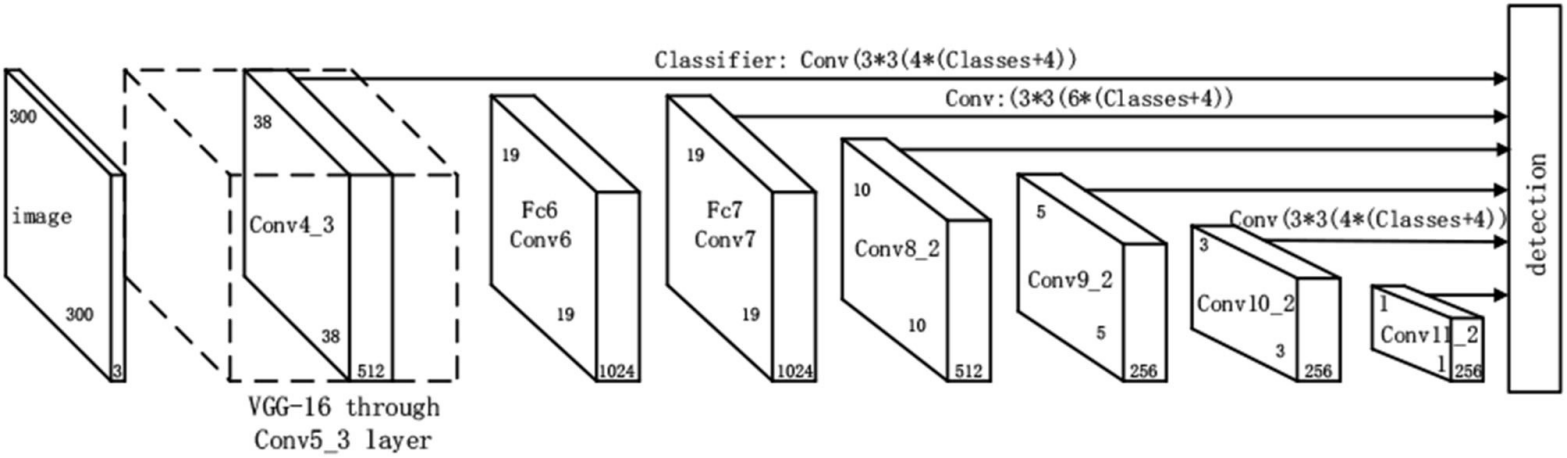
SSD network structure.

SSD algorithm is a single-stage target detection method, which can complete target identification and location tasks in one step and has a fast detection speed (Luo et al., 2020; Sun et al., 2020c; Yang et al., 2021). In addition, SSD network combines YOLO's regression idea and FtP-RCNN's anchor boxes mechanism to predict multi-scale target objects by using prior boxes of different numbers and sizes on feature maps of different scales. Prior box is an anchor frame that traverses feature maps with sliding windows of different sizes and generates different lengths, widths, and aspect ratios. Figure 1 shows the SSD network model (Sun et al., 2020c).

SSD network adopts VGG16 as the main dry network (Tan et al., 2020; Yun et al., 2022), and converts full-connection layer FC6 and FC7 of VGG16 into a convolution layer Conv6 of 3 ×3 and convolution layer Conv7 of 1 ×1 respectively. Meanwhile, pool5 is changed from 2 ×2 of original stride=2 to 3 ×3 of stride = 1. The corresponding Conv6 uses extended convolution or Dilation Conv to enlarge the field of convolution. At the same time, SSD network adopts Convolution of Stride =2 to reduce the size of the feature graph, thus obtaining the feature graph of different sizes Conv4_3, Conv7, Conv8_2, Conv9_2, Conv10_2, and Conv11_2, and the detection result is obtained by convolution of the feature graph of each layer. The detection results include category confidence and bounding box position. SSD network adopts anchor mechanism, and the prediction box is obtained by non-maximum suppression method on the basis of the prior box. The so-called non-maximum suppression is to sort the confidence score of the target category, select the prior box with the highest confidence, calculate the union ratio between the boundary box with the highest confidence and other candidate boxes, delete the prior box with the union ratio greater than the threshold value, and then delete the redundant prior box to generate the final prediction box. The setting of prior box includes two aspects: size and aspect ratio. As for the size of prior box, as the feature graph decreases, its receptive field increases, and the size of the corresponding prior box increases linearly, as shown in the formula below.


(1) $$\begin{array}{r} S_{k}=S_{\min}+\frac{S_{\max}-S_{\min}}{m-1}\left(k-1\right),\;k\in \left[1,m\right] \end{array}$$

In which, *S*_*k*_ represents prior box size relative to the proportion of the input image, and *S*_max_ and *S*_min_ represent the maximum value 0.9 and the minimum value 0.2 of the proportion; *m* is the number of feature graphs, with a value of 5, because the prior box size of Conv4_3 layer was set as D separately. The following feature graphs were linearly increased according to the above formula, but the scale was expanded 100 times first, and the growth step was as follows:


(2) $$\begin{array}{r} \left[\left(S_{\max}\times 100\right)-\left(S_{\min}\times 100\right)\right]/\left(m-1\right)=17 \end{array}$$

In this way, the *S*_*k*_ of each feature graph is 20, 37, 54, 71, 88, and 105, respectively. These ratios are divided by 100 and then multiplied by the image size to get the size of each feature graph as 30, 60, 111, 162, 213, 264, and 315. Thus, the minimum size and maximum size of the prior box generated by each feature layer are shown in Table 1.

**Table 1:** Prior box size of each feature layer.

**Feature_map**	**Number of prior boxes**	**Minimum size**	**Maximum size**
Conv4_3	4	30	60
Fc7(Conv7)	6	60	111
Conv8_2	6	111	162
Conv9_2	6	162	213
Conv10_2	4	213	264
Conv11_2	4	264	315

Length to width ratio is generally selected as *a*_*r*_∈{1, 2, 3, 1/2, 1/3}. After the aspect ratio is determined, the width and height of the prior box are calculated according to the following formula, which is the actual size of the prior box:


(3) $$\begin{array}{r} w_{k}^{a}=S_{a}\sqrt{a_{r}},\;h_{k}^{a}=S_{a}/\sqrt{a_{r}} \end{array}$$

By default, each feature graph will have an a priori box of size *S*_*a*_ and *a*_*r*_ = 1. In addition, an a priori box with scale $S_{a}^{{}^{\prime }}=\sqrt{S_{a}S_{a+1}}$ will be set, and *a*_*r*_ = 1. Therefore, each feature graph is set with two square a priori boxes of different sizes. The center point of the priori box of each cell is distributed in the center of each cell, that is, the coordinate is (*i*+0.5/|*f*_*k*_|, *j*+0.5/|*f*_*k*_|), *i, j*∈[0, |*f*_*k*_|], where |*f*_*k*_| is the size of the feature graph.

After obtaining the a priori box, you need to determine which a priori box matches the real target, that is, the boundary frame corresponding to the a priori box matching the real target will be responsible for predicting it. There are two main matching principles between the a priori box of SSD and the real target: (1) for each target in the picture, find the a priori box with the largest intersection ratio, and the a priori box will match it. An a priori box that matches the target is usually called a positive sample. On the contrary, if an a priori box does not match any target, the a priori box is a negative sample. There are a few targets in a picture relative to the background, so the generated a priori box is prone to imbalance between positive and negative samples; and (2) on the basis of principle (1), for the remaining unmatched a priori box, if the intersection and union ratio of a real target is greater than a certain threshold (generally 0.5), the a priori box is also matched with the real target, that is, a target can have multiple a priori boxes, and each a priori box can only match one target. If the intersection and union ratio of multiple targets with an a priori box is greater than the threshold, the a priori box can only match the target with the largest intersection and union ratio. In addition, in order to reduce the impact of the imbalance of positive and negative samples, SSD network samples the negative samples according to the confidence, and selects the Top-k with large error as the training negative sample to ensure that the proportion of positive and negative samples is close to 1:3.

The loss function of SSD network is defined as the weighted sum of position error and confidence error.


(4) $$\begin{array}{r} L\left(x,c,l,g\right)=\frac{1}{N}\left(L_{conf}\left(x,c\right)+\alpha L_{loc}\left(x,l,g\right)\right) \end{array}$$

In which,


(5) $$\begin{array}{r} L_{loc}\left(x,l,g\right)=\overset{N}{\underset{i\in Pos}{\sum }}\underset{m\in \left\{cx,cy,w,h\right\}}{\sum }x_{ij}^{k}smooth_{L1}\left(l_{i}^{m}-{\overset{\wedge }{g}}_{j}^{m}\right) \end{array}$$

N is the number of positive samples in the a priori box, *c* is the predicted value of category confidence, *l* is the position prediction value of the corresponding boundary box of the a priori box, *g* is the position parameter of the real target, and ${\overset{\wedge }{g}}_{j}^{cx}$ is the encoding of the real box; the weight coefficient α is set to 1 through cross validation; $x_{ij}^{p}\in \left\{0,\;1\right\}$ is an indication parameter. When $x_{ij}^{p}=1$, it means that the a priori box *i* matches the target *j*; *p* is the category of the target. For the confidence error, it adopts the Softmax loss function, which is defined as follows:


(6) $$\begin{array}{l} L_{conf}\left(x,\;c\right)-\sum_{i\in Pos}^{N}x_{ij}^{p}\log{\hat{c}}_{i}^{p}-\sum_{i\in Neg}\log{\hat{c}}_{i}^{o},\;where\\ \;{\hat{c}}_{i}^{p}=\frac{\exp\left(c_{i}^{p}\right)}{\sum_{p}\exp\left(c_{i}^{p}\right)} \end{array}$$

An SSD network figure design according to the characteristics of the different sizes and using the different scales of maps to improve the accuracy of the single-phase target detection algorithm, will realize the balance between speed and accuracy (Huang et al., 2021; Jiang et al., 2021a). But it only uses a single measure of the characteristics of the figure of target detection. This means detection of target scale has certain limitations; without considering the complementarity and relevance of multi-scale features, it is easy to have problems of inaccurate positioning and high classification error rate, and the insufficient feature semantic information used to detect small targets is easy to lead to small target shoulder (He et al., 2019; Chen et al., 2021b; Xu et al., 2022). Therefore, the detection accuracy of this algorithm still has room for improvement.

### SSD Target Detection Algorithm Based on Multi-Scale Feature Fusion

**Figure 2:**
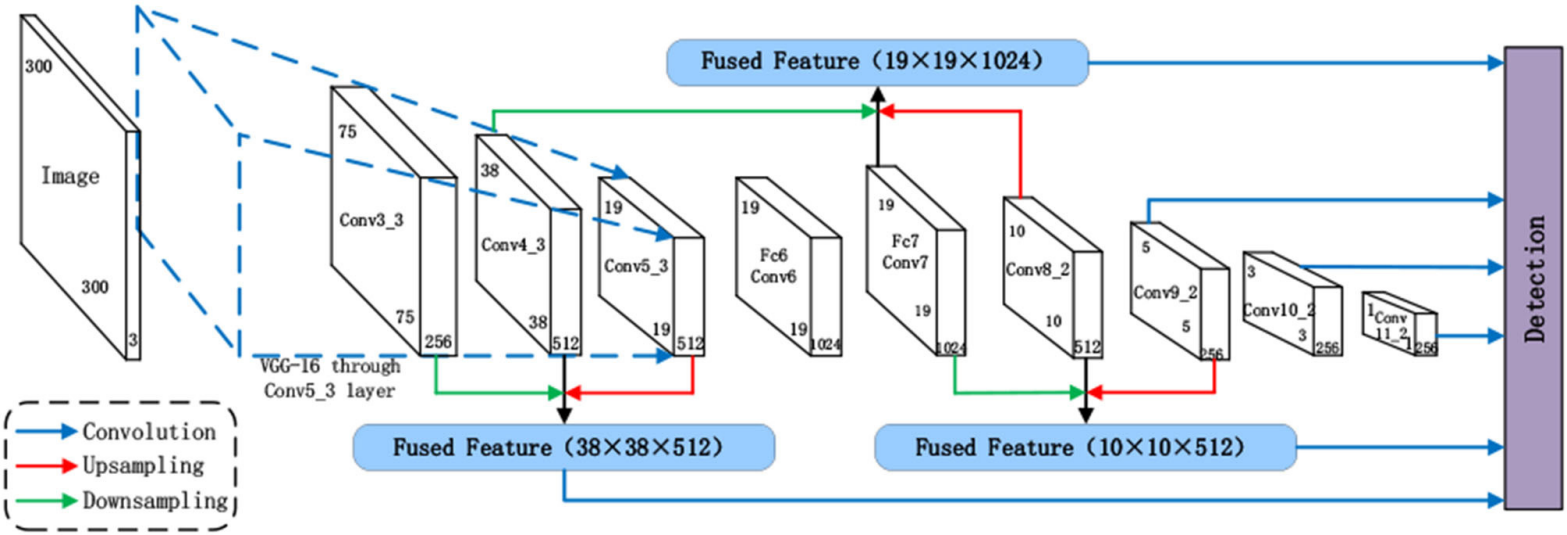
SSD network structure based on multi-scale feature fusion.

In order to improve the detection accuracy of SSD target detection algorithm in actual complex scenes and promote the application of target detection technology in service robots, a multi-scale feature fusion algorithm was proposed in this paper. The features of prediction layer and adjacent layer were fused for detection. For Conv7, in order to make full use of low-level location information, it was fused with Conv4_3 feature map. The improved SSD network not only makes full use of multi-scale features, but also enhances the complementarity of high- and low-level features, improves the detection performance of SSD network for multi-scale targets, and improves the practicality of the model in complex scenarios. The SSD network structure based on multi-scale feature fusion is shown in Figure 2.

In order to ensure the invariable size of the feature map of the target prediction layer and prevent the problems of large spatial resolution of the feature after fusion, large difference in information distribution from the high-level feature map, and difficulty in learning the network in the later stage, the target prediction layer is taken as the benchmark and the features of its adjacent layers, namely features of different scales, are detected after fusion. The feature maps lower than the prediction layer are down-sampled, while the adjacent higher-level features are up-sampled. Through the effective fusion of multi-level and multi-scale target feature information, the feature layer used for prediction can make full use of multi-scale and multi-level target features, improve the detection ability of multi-scale targets, and improve the overall detection performance.

Feature extraction and fusion methods have a great influence on the prediction of late-stage features. In order to make full use of multi-scale features at all levels and reduce the influence of network improvement on late-stage data processing, the extraction and fusion methods of multi-scale features at all levels are designed in this section. It is necessary to ensure the consistency of resolution of feature maps before integrating features of different scales. Taking Conv4_3 as an example, the processing method of Conv3_3 and Conv5_3 adjacent features is further explained. Among them, the features that are lower than the target detection layer are called low-level features, and the features that are lower than the target detection layer are called high-level features.

**Figure 3:**
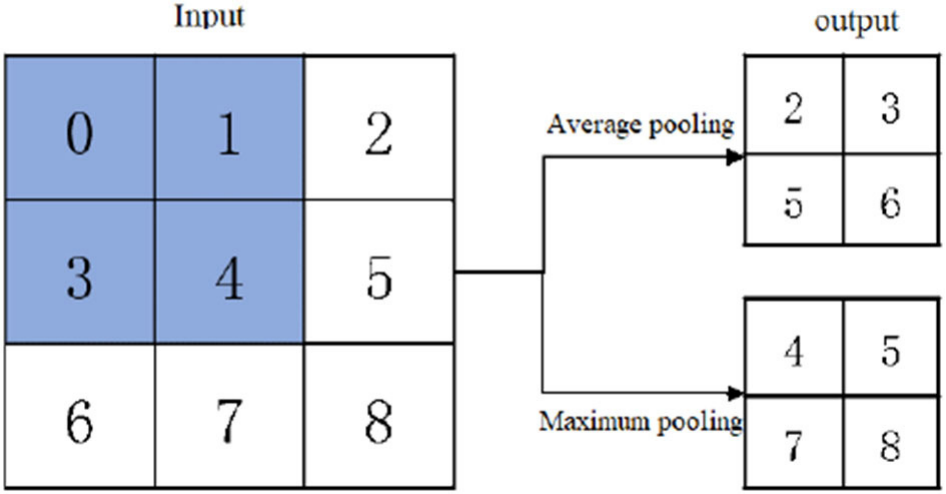
Average pooling and maximum pooling.

The commonly used down-sampling methods include maximum pooling and average pooling. Maximum pooling slides on the feature graph in the form of a window and selects the maximum value of the window area on the feature graph as the output eigenvalue, while average pooling takes the mean value as the output. As shown in Figure 3, the inputs of convolution check are averaged and maximized respectively to obtain corresponding outputs. Compared with average pooling, the overall characteristics of data can be better preserved, while maximum pooling can preserve texture information better. Therefore, maximum pooling was selected to down sample Conv3_3. However, the direct maximum pooling of Conv3_3 would lead to the loss of partial position and detail features of Conv3_3, so the feature extraction of Conv3_3 was carried out by 3 ×3 convolution first, and then the maximum pooling was carried out.

**Figure 4:**
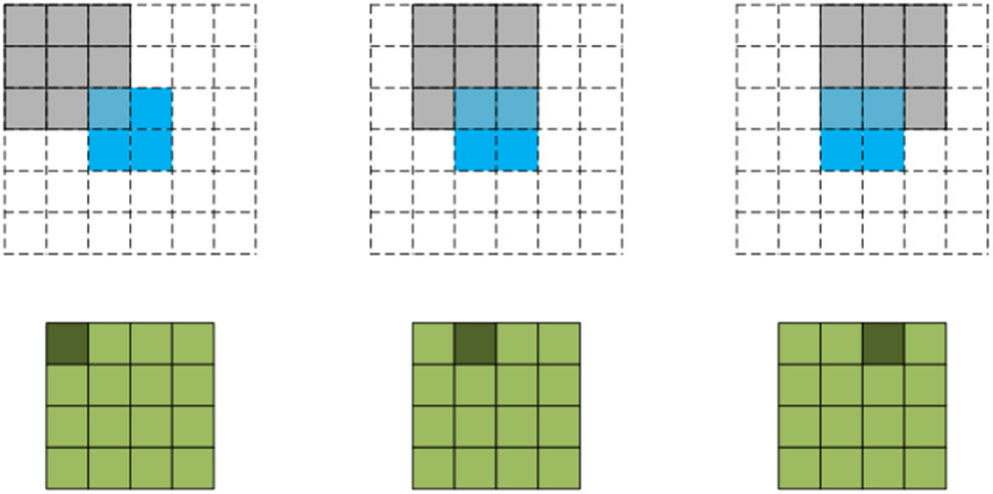
Deconvolution up-sampling.

Common up-sampling methods are bilinear interpolation and transpose convolution, also known as deconvolution. Deconvolution is shown in Figure 4, for the input of2 ×2, the convolution kernel of 3 ×3 is adopted with step *stride* = 1, and the input boundary is filled with *padding* = 2. The green output corresponding to the next line 5 ×5 is obtained by traversing the filled feature graph. Compared with bilinear interpolation, deconvolution up-sampling is more flexible and can extract features effectively. Therefore, deconvolution is used to up-sample high-level features in this paper, and high-level features are normalized before fusion, making the network easier to train and alleviating the gradient dispersion problem of deep network to a certain extent.

**Figure 5:**
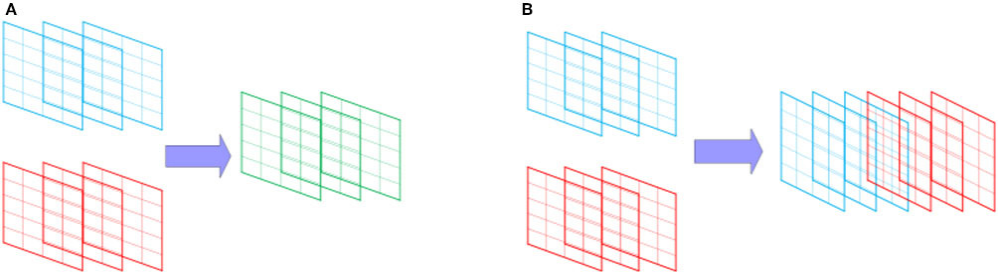
Different methods of feature fusion. **(A)** Element-by-element addition, **(B)** cascade.

The commonly used feature fusion methods include cascade and element-by-element addition. Figure 5 shows two different feature fusion methods, in which addition means directly adding the pixel values corresponding to the features of each layer. The dimensions of the features before fusion are consistent, while the dimensions of the features after fusion remain unchanged. Cascade does not require the dimension of features before fusion, but requires the same dimension of features, and features after cascade need to adjust the dimension of features by using convolution.

**Figure 6:**
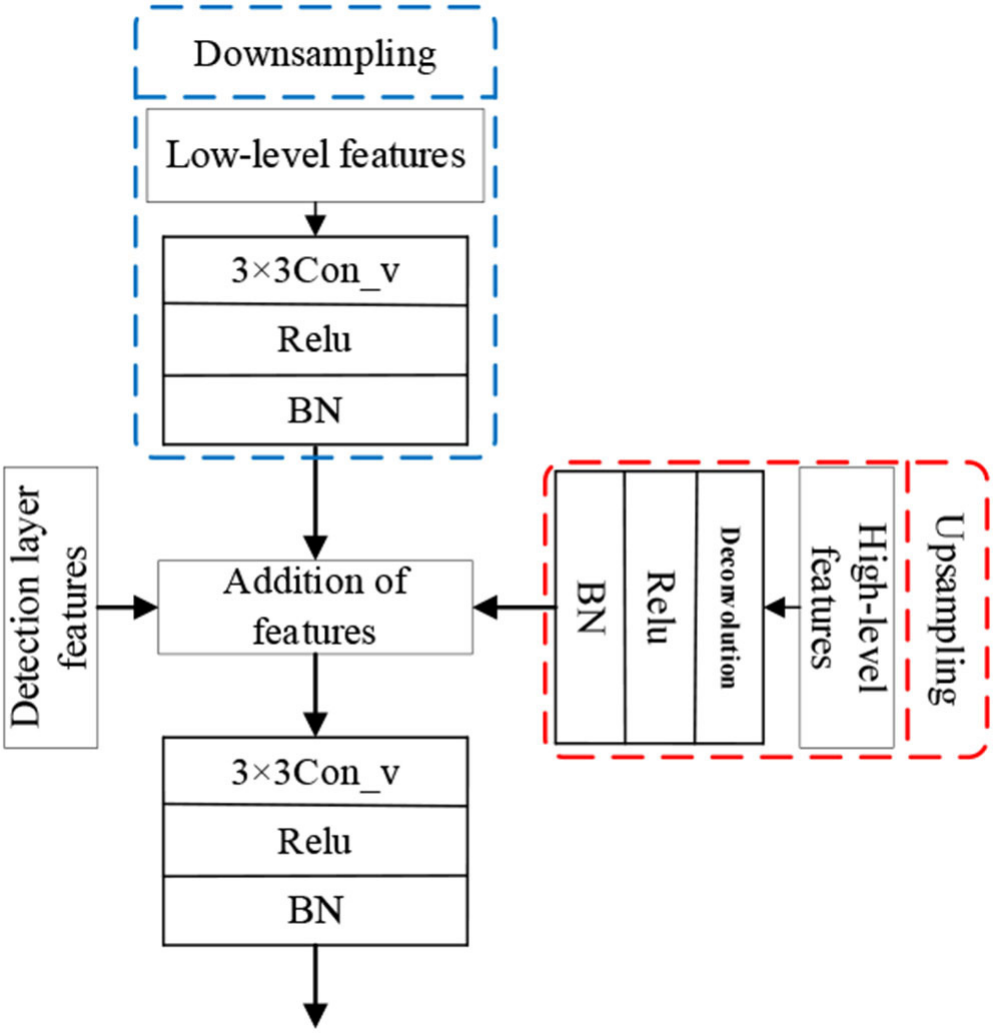
Multi-scale feature fusion structure.

For feature fusion method, considering the target detection layer dominated, and other characteristics of the layer, and adopting the way of cascade fusion features easy to the expansion of the dimension problem, therefore, this article adopts the method of pixel addition to multi-scale feature fusion, and the characteristics of the fused 3x3 convolution to reduce feature fusion after stack effect. The difference of feature distribution among feature maps at all levels is eliminated and the information of feature maps at all levels are fused. Thus, the multi-scale feature fusion module is obtained as shown in Figure 6, and the same fusion structure is adopted for the multi-scale feature fusion of other target detection layers.

## Data set Establishment and Experiment Based on Indoor Environment

### Establishment of Indoor Scene Data set

**Figure 7:**
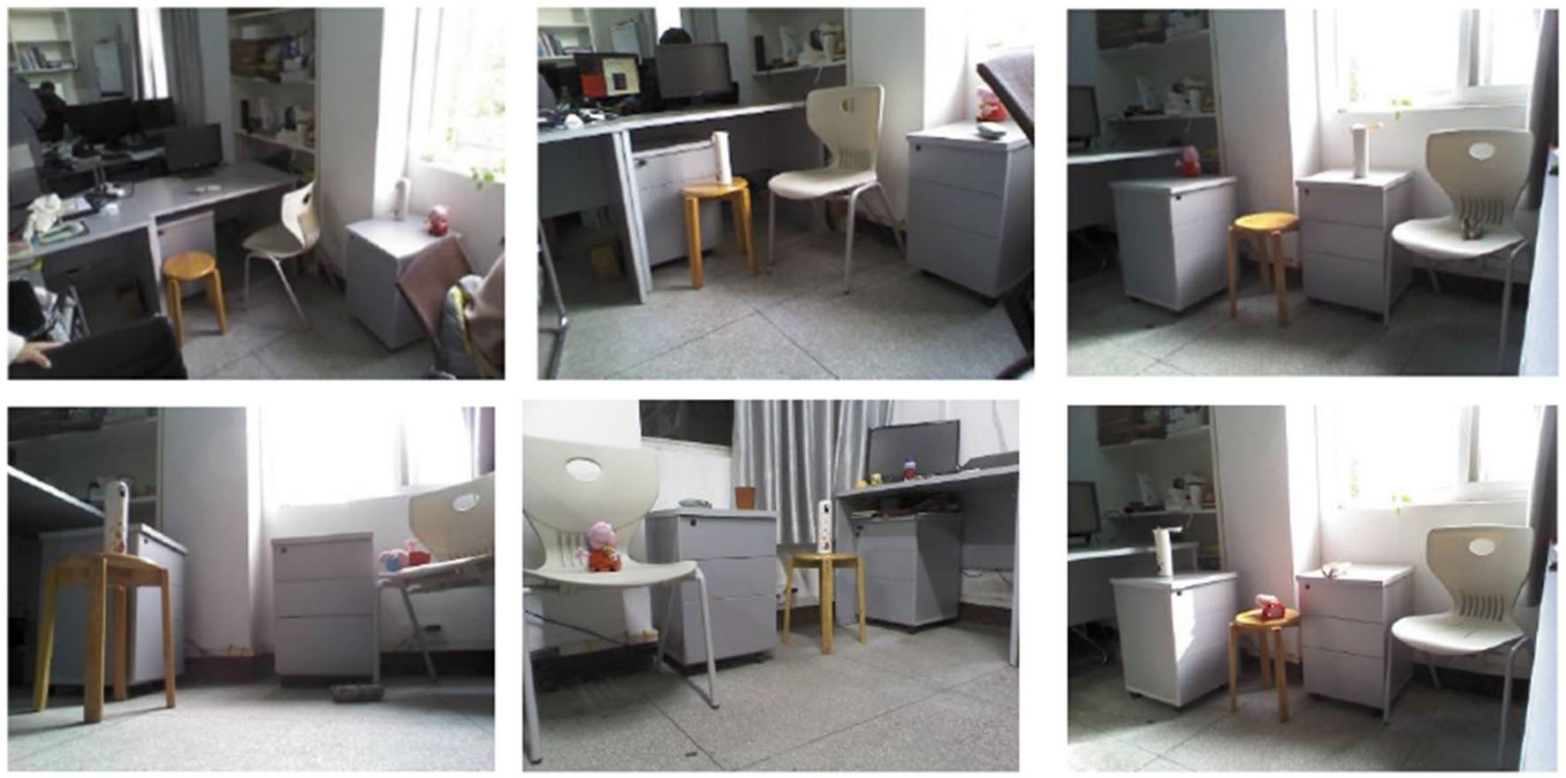
Color images from different angles, backgrounds, and illumination.

Common objects in daily life are used as detection targets, including toys, chairs, stools, cabinets, glasses, cases, and cups. In the process of image collection, 1064 studio interior scene color images of different backgrounds, different light intensities, and different angles were collected considering the complex scene background, occlusion between the target objects, lighting, and angle changes. At the same time, the deformation of toy, vacuum cup, glasses case, and the shape similarity of chair and stool were considered to improve the robustness of the target detection model, as shown in Figure 7. The collected pictures are named with four Arabic numerals in a one-to-one correspondence.

In deep learning, in order to obtain a better model, a large amount of data is needed to fully extract and learn the feature information of the target to achieve better detection accuracy and robustness. When the amount of data is too small, the network model learning is insufficient and difficult to converge, or the network is too dependent on the existing data and lacks flexibility. In addition, for a target detection task, in order to prevent the model from having a good detection effect on some objects but a poor detection effect on other objects, the model needs a good generalization. The model is also required to be fully learned for each category of objects, so the number of samples for each category should be as balanced as possible. Data enhancement technique for this block provides a solution; the diversity of the data generation of data to enhance the use of the existing value of the data, for example, random adjustment of chromaticity in a deep learning neural network will not only be based on the color information of object recognition, but will learn the typical semantic information of target objects.

**Figure 8:**
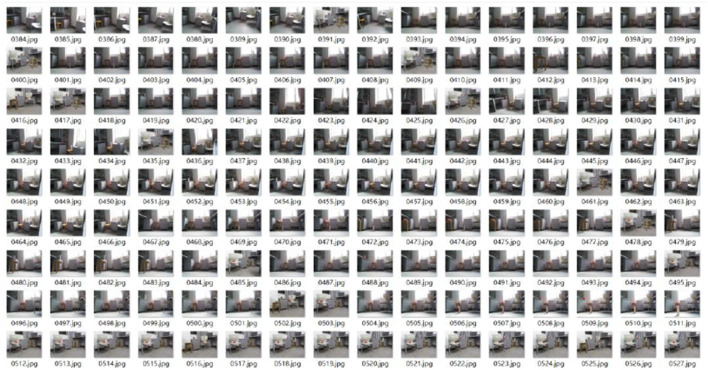
Partial images of target detection dataset in a complex indoor scene.

Although the color images collected in the complex indoor scene in this paper have considered multiple situations of target detection in the application of indoor service robots as much as possible, due to the limited site and resources, the data still cannot meet the diverse needs of practical application. Therefore, in order to increase the robustness of the model and improve the ability of the model to resist noise interference, random image processing is carried out on the collected image data in this paper to enrich the samples and improve the detection performance and generalization of the trained model. Under the condition that other conditions remain unchanged, the following operations are carried out on the collected images to expand the data set to 4256, so as to realize the creation of the target detection data set of complex indoor scenes. The indoor scene image data set constructed from this is shown in Figure 8.

**Figure 9:**
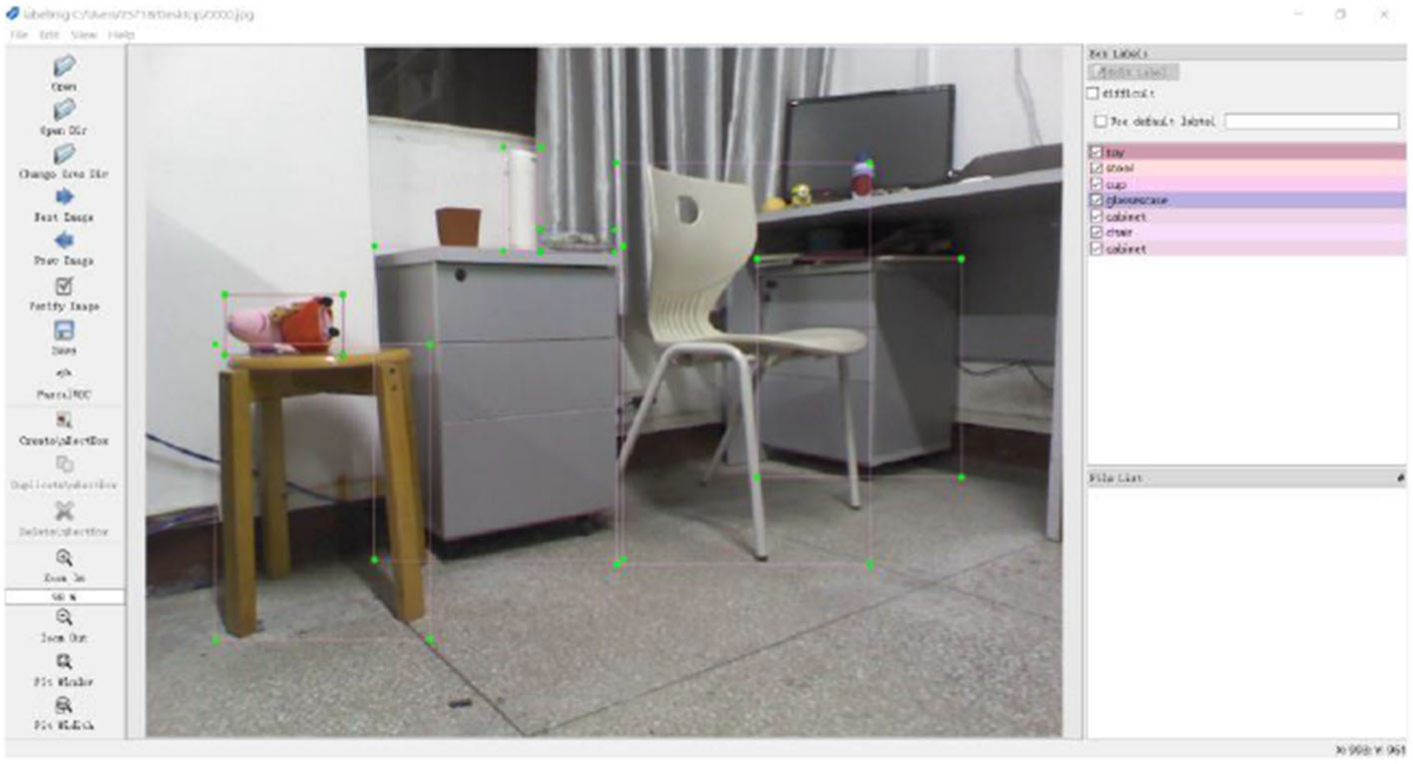
LabelImg annotation interface.

LabelImg software was used to manually mark the categories and corresponding positions of the target objects contained in the image. Before annotating data, you need to pre-define its category for later annotation. The category information in this paper includes toy, chair, stool, cabinet, glasses case, cup, and others in the background. First, double-click to open the labelImg. Extract the file and set the file path read and store it in the upper left corner. The path cannot contain Chinese characters. Mark the position of each target object according to the standard of minimum enveloping rectangular box and select the corresponding category, thus completing the target detection labeling of image data, as shown in Figure 9.

### Experiment and Analysis

In order to select the detection model with the best application effect in the actual indoor scene, this paper uses the constructed indoor scene data set to conduct experiments on the Faster R-CNN, YOLOv5, SSD, and SSD algorithm based on multi-scale feature fusion, and compares the detection performance of each network. The parameter configuration of the experimental environment is shown in Table 2.

**Table 2:** Related parameters of experimental environment.

**Category name**	**Detail**
Operating system	Windows10
CPU	AMD Ryzen 7
GPU	NVIDIA GeForce RTX 2070
Cuda with Cudnn	10.0/7.6.5
Python	3.6
Tensorflow, Keras	1.13.2/2.1.5
Opencv	4.5.1

Firstly, feature fusion is performed layer by layer for SSD target detection layer to obtain the optimal SSD target detection model based on multi-scale feature fusion. In the training process, the pre-training file of the model on PASCAL VOC data set was used to initialize the weights, and the weight training and detection network of the trunk network was frozen, and then the weight of the trunk network was unfrozen to train the model, that is, the idea of transfer learning was used to accelerate the model training speed. The average accuracy and overall accuracy of each item detected by layer feature fusion are shown in Table 3.

**Table 3:** Comparison of detection performance of feature fusion networks at different target detection layers.

**Item category**	**Stool**	**Chair**	**Cabinet**	**Toy**	**Cup**	**Glasses box**	**mAP (%)**
**Fusion layer**							
No fusion	0.9838	0.9898	0.9658	0.9121	0.9046	0.4715	87.13
Conv3_3/Conv4_3/Conv5_3	0.9855	0.9889	0.9638	0.9823	0.9757	0.8115	95.13
Conv4_3/Conv7/Conv8_2	0.9936	0.9910	0.9902	0.9118	0.8953	0.4819	87.73
Conv7/Conv8_2/Conv9_2	0.9822	0.9895	0.9626	0.9195	0.8926	0.4841	87.17
Multi-scale feature fusion	0.9980	0.9990	0.9970	0.9947	0.9841	0.8513	96.90

As shown in Table 3, the overall detection accuracy of the SSD algorithm is 87.13%, and the detection effect is poor for the relatively small size of the eyeglass case, thus reducing the effect of the whole detector. However, it is inevitable that the target scale varies in the actual application scenarios, so the detection effect of each scale target needs to be improved. According to the comparison of detection accuracy before and after multi-scale feature fusion in Table 3, multi-scale feature fusion of different target detection layers can improve the detection effect of small objects, and compared with the other two fusion methods, the fusion Conv3_3, Conv4_3, and Conv5_3 has better detection effect on multi-scale targets. Feature fusion is performed on all target detection layers, and the detection accuracy of SSD network is greatly improved. MAP can reach 96.90%, which verifies the effectiveness of multi-scale feature fusion method.

**Figure 10:**
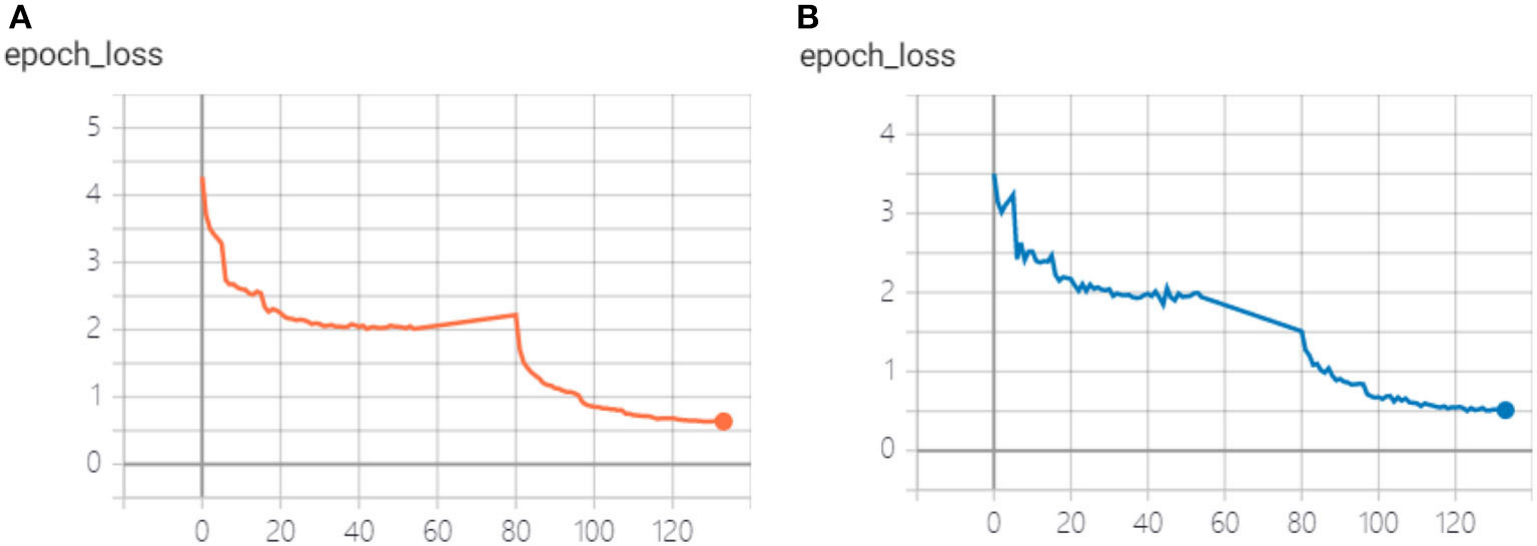
SSD network training based on multi-scale feature fusion. **(A)** Loss of training set, **(B)** loss of verification set.

In the indoor scenario data set, we train Faster R-CNN, YOLOv5, SSD, and SSD network based on multi-scale feature fusion using transfer learning idea. The training process of SSD network based on multi-scale feature fusion is shown in Figure 10. During the training, the weight freezing was first iterated 80 times, but the loss of the detection model tended to converge after about 45 times. Then the weight unfreezing was used to train the whole network, and the model training was completed after more than 120 iterations.

Table 4 shows the performance test results of each algorithm on the indoor scene data set. As can be seen from the table, in terms of detection accuracy, Faster R-CNN is the best, followed by SSD algorithm, and YOLOv5 is the worst but the fastest. The accuracy of SSD algorithm based on multi-scale feature fusion is greatly improved compared with SSD, but the detection speed is decreased, but it is still higher than Faster R-CNN.

**Table 4:** Test results of different network models on data sets.

**Algorithm**	**Precision (mAP, %)**	**Detection speed (FPS)**
Faster R-CNN	98.78	12
YOLOv5	82.83	36
SSD	87.13	26
SSD with multi-scale feature fusion	96.90	19

**Figure 11:**
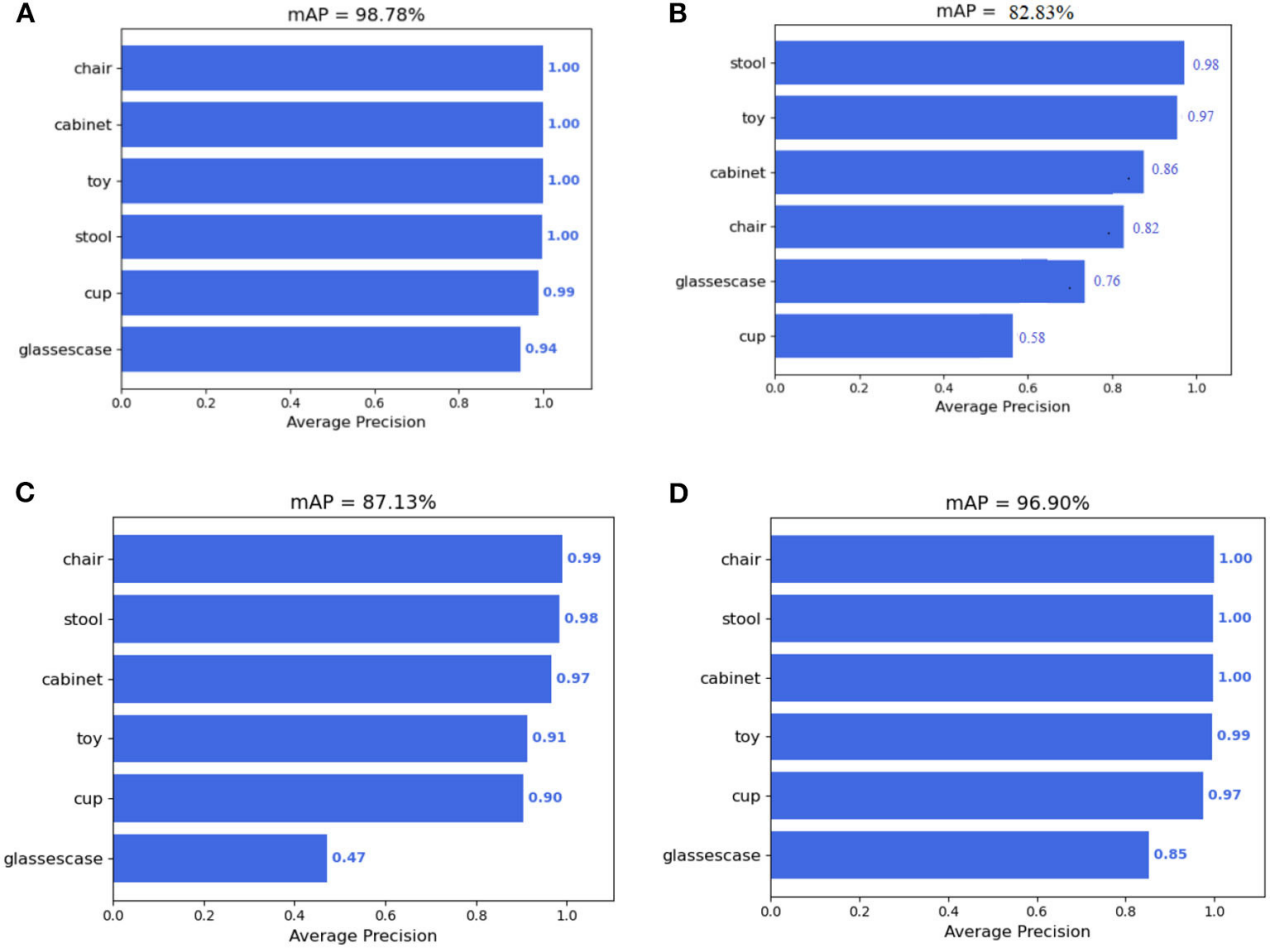
Detection of each algorithm corresponding to different classes in the indoor scene. **(A)** Faster R-CNN. **(B)** YOLO v5. **(C)** SSD. **(D)** SSD network with multi-scale feature fusion.

**Figure 12:**
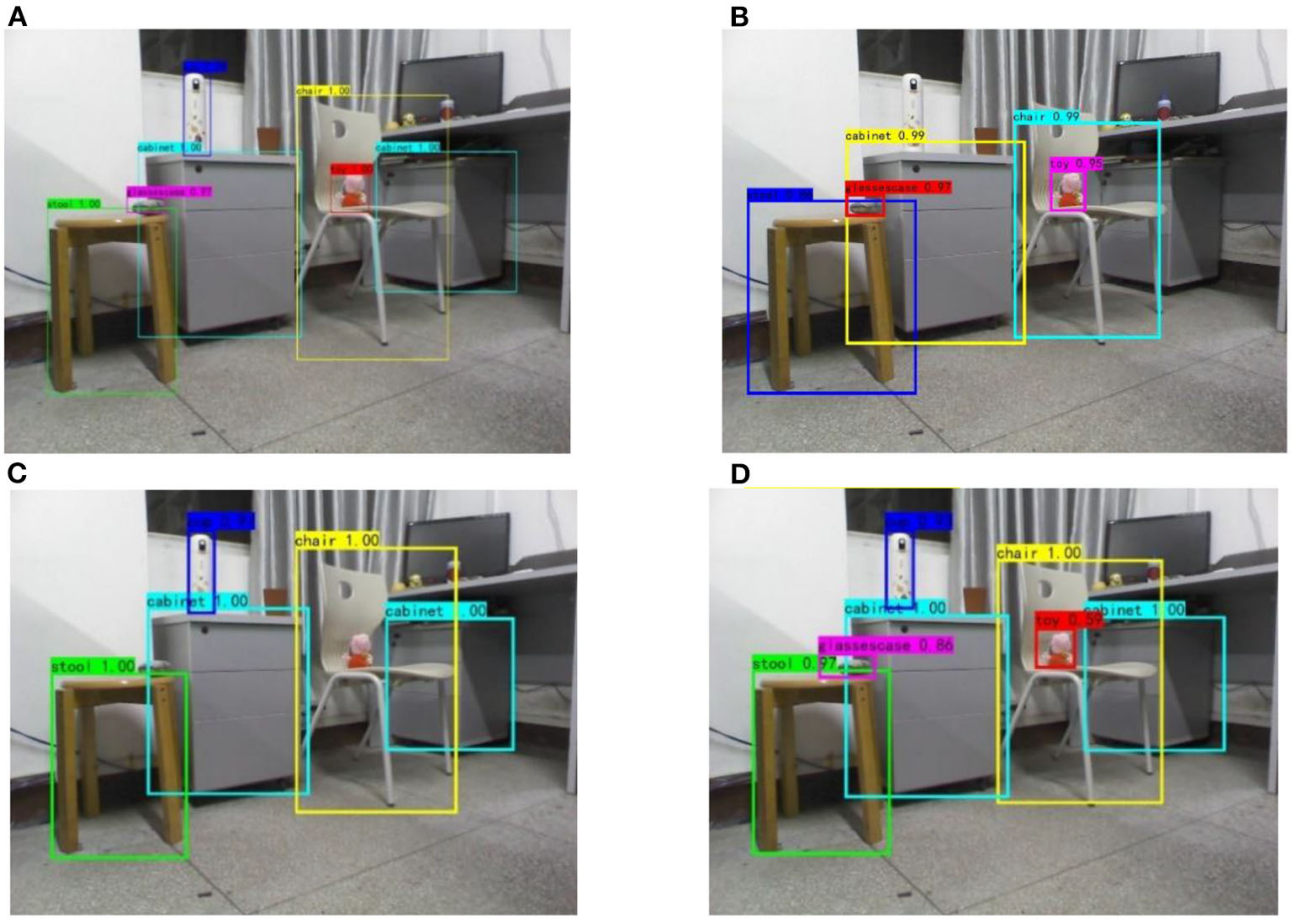
Comparison of detection effects of various networks. **(A)** Faster R-CNN. **(B)**YOLO v5. **(C)** SSD. **(D)** SSD network with multi-scale feature fusion.

The detection accuracy of several algorithms for various objects is shown in Figure 11. When the confidence threshold is 0.5, the detection effect of several algorithms under the same image is shown in Figure 12. It can be seen from the figure that Faster R-CNN has a good detection effect on all targets, while YOLOv5 has misjudgment on cups and cabinets with similar backgrounds due to the lack of detailed information in multiple convolutions, and the detection frames of all objects are inaccurate. And SSD based on multi-scale feature fusion is the SSD, to the detection effect of the target objects are promoted, which for the ascension of glasses box, cup, and toy effect is more apparent, and verify the SSD based on multi-scale feature fusion algorithm for multiscale target for testing the effectiveness of conforming to the requirements of the indoor service robot application.

## Conclusion

In order to solve the problem of low detection accuracy and poor effect of small target detection technology, this paper proposes a service robot target detection method based on multi-scale feature fusion. The SSD algorithm with fast detection speed is improved, and the features of its target detection layer are fused with adjacent features for detection. Full use of the complementarity between different levels of features and the correlation between multi-scale features is made. The feature fusion of the target detection layer by layer was carried out in the self-built indoor scene target detection data set using the transfer learning idea, and the comparison experiment was conducted with Faster R-CNN, YOLOv5, and SSD. Experimental results show that the fusion of multi-scale features greatly improves the detection accuracy of SSD algorithm, and the improvement effect is more obvious for small scale objects. In addition, compared with the YOLO algorithm, the improved SSD algorithm has a higher detection accuracy, while compared with the Faster R-CNN, the improved SSD algorithm has a faster detection speed. The SSD algorithm based on multi-scale feature fusion achieves a better balance between target detection accuracy and detection speed. In this paper, the method of multi-scale feature fusion is used to enhance the semantic feature expression of multi-scale targets and small objects. In the subsequent research on target detection, attention mechanism can be introduced to improve the network to promote the effective learning of features by the model and improve the detection performance of the algorithm.

## Data Availability Statement

The original contributions presented in the study are included in the article/supplementary material, further inquiries can be directed to the corresponding author/s.

## Author Contributions

LH and CC provided research ideas and plans. CC and JY wrote programs and conducted experiments. LH, ZH, and JT analyzed and explained the simulation results. CC and YS improved the algorithm. HY co-authored the manuscript. HY and HM were responsible for collecting data. JY and HM revised the manuscript for the corresponding author and approved the final submission. All authors contributed to the article and approved the submitted version.

## Funding

This work was supported by grants from the National Natural Science Foundation of China (Grant Nos. 52075530, 51575407, 51975324, 51505349, 61733011, and 41906177), the Grants of Hubei Provincial Department of Education (D20191105), the Grants of National Defense PreResearch Foundation of Wuhan University of Science and Technology (GF201705), Open Fund of the Key Laboratory for Metallurgical Equipment and Control of Ministry of Education in Wuhan University of Science and Technology (2018B07 and 2019B13), and the Open Fund of Hubei Key Laboratory of Hydroelectric Machinery Design and Maintenance in China Three Gorges University (2020KJX02 and 2021KJX13).

## Conflict of Interest

The authors declare that the research was conducted in the absence of any commercial or financial relationships that could be construed as a potential conflict of interest.

## Publisher's Note

All claims expressed in this article are solely those of the authors and do not necessarily represent those of their affiliated organizations, or those of the publisher, the editors and the reviewers. Any product that may be evaluated in this article, or claim that may be made by its manufacturer, is not guaranteed or endorsed by the publisher.
